# Precision Mapping of COVID-19 Vulnerable Locales by Epidemiological and Socioeconomic Risk Factors, Developed Using South Korean Data

**DOI:** 10.3390/ijerph18020604

**Published:** 2021-01-12

**Authors:** Bayarmagnai Weinstein, Alan R. da Silva, Dimitrios E. Kouzoukas, Tanima Bose, Gwang Jin Kim, Paola A. Correa, Santhi Pondugula, YoonJung Lee, Jihoo Kim, David O. Carpenter

**Affiliations:** 1 Department of Environmental Health Sciences, School of Public Health, University at Albany, Rensselaer, New York, NY 12144, USA; bmunkhjargal@albany.edu; 2 Principles and Practice of Clinical Research Program, T.H. Chan School of Public Health, Harvard University, Boston, MA 02115, USA; 3 Department of Statistics, University of Brasília, Brasília 70910-900, Brazil; alansilva@unb.br; 4 Research Service, Edward Hines Jr. VA Hospital, Hines, IL 60141, USA; dimitrios.kouzoukas@va.gov; 5 Department of Molecular Neuroscience and Pharmacology, Loyola University Chicago, Maywood, IL 60153, USA; 6 Institute for Clinical Neuroimmunology, Ludwig-Maximilian University of Munich, Planegg-Martinsried, 82152 Munich, Germany; tanima.bose@med.uni-muenchen.de; 7 Institute of Experimental and Clinical Pharmacology and Toxicology, Faculty of Medicine, University of Freiburg, 79104 Freiburg, Germany; gwang-jin.kim@pharmakol.uni-freiburg.de; 8 Howard Hughes Medical Institute, Ashburn, VA 20147, USA; correap@janelia.hhmi.org; 9 Department of Pharmacology & Therapeutics, University of Florida, Gainesville, FL 32610, USA; psanthi@ufl.edu; 10 Department of Pharmaceutical Sciences, School of Pharmacy, Texas Tech University Health Sciences Center, Amarillo, TX 79106, USA; yoonjung.lee@ttuhsc.edu; 11 Department of Computer Science, Hanyang University, Seongdong-gu, Seoul 04763, Korea; datartist@agape.hanyang.ac.kr; 12 Institute for Health and the Environment, University at Albany, Rensselaer, NY 12144, USA

**Keywords:** COVID-19, pandemics, socioeconomic factors, spatial regression, South Korea

## Abstract

COVID-19 has severely impacted socioeconomically disadvantaged populations. To support pandemic control strategies, geographically weighted negative binomial regression (GWNBR) mapped COVID-19 risk related to epidemiological and socioeconomic risk factors using South Korean incidence data (20 January 2020 to 1 July 2020). We constructed COVID-19-specific socioeconomic and epidemiological themes using established social theoretical frameworks and created composite indexes through principal component analysis. The risk of COVID-19 increased with higher area morbidity, risky health behaviours, crowding, and population mobility, and with lower social distancing, healthcare access, and education. Falling COVID-19 risks and spatial shifts over three consecutive time periods reflected effective public health interventions. This study provides a globally replicable methodological framework and precision mapping for COVID-19 and future pandemics.

## 1. Introduction

COVID-19 has corroborated insights gained from SARS, H1N1 influenza, and MERS pandemics showing socioeconomically disadvantaged populations are more severely impacted from pandemics [1,2,3,4]. The reported gap between the COVID-19 rates of the most and least advantaged populations [5] present a potential for reducing the outbreak through targeted interventions. Unlike non-modifiable factors [6,7], such as populations with genetic predispositions, population vulnerability to the infectious disease outbreaks can be remediated through targeted interventions. Population vulnerability to respiratory infectious diseases is characterized by multiple interrelated factors, such as family income, education, employment status, health behaviour, healthcare access and other area-health indicators [8,9]. Hence, identifying key socio-economic determinants for COVID-19 and mapping the vulnerable locales will enable policy makers to target specific modifiable factors in high-risk areas.

Among various approaches to disentangle how socio-economic status (SES) impacts health, Coleman’s social theory has been regarded as exceedingly useful because of its treatment of SES beyond access to material resources but also a function of social and human capital that ‘*uniquely locate the individual’s status in the social structure*’. Blumenshine furthered the understanding by illustrating the mechanistic pathways between the socio-economic position and health disparity, as the underlying socioeconomic determinants of individuals can determine their likelihood of being exposed to the pandemic virus, contracting disease and timely and effective treatment after the disease developed [9].

**Figure 1:**
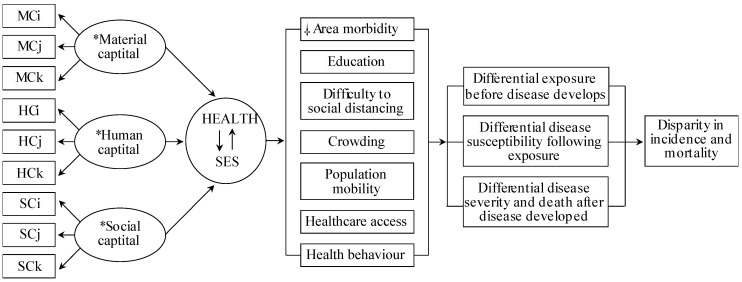
Conceptual model of the causal relationship between the SARS-CoV-2 and area health/SE determinants. Abbreviations: material capital (MC), human capital (HC), social capital (SC), socioeconomic status (SES). Subscripts (i, j, k) indicate the number of variables used from the data sources. * Material, Human and Social Capital refers to latent structural components of the SES and COVID-19-specific determinants. Health and SES connected by arrows indicate the inter-relatedness of area health and SES, hereinafter, denoted as a health/SE. ⸸ Area-health/SE themes identified relevant to COVID-19 based on the current person and population-level literature. As per Coleman’s social theory and contributing data underlying each health/SE theme, crowding, healthcare access and social distancing relates to material capital, health behaviour and area morbidity relates to human capital, whereas, crowding, education and population mobility to social capital. Modified from source [9,24]. Data sources: Korean Community Health Survey by the KCDC, Health Insurance Statistics by the National Health Insurance Services, Disability Status by the Ministry of Health and Welfare, Death Cause Statistics by the National Statistics Agency, Korean Census Bureau, Internal Migration Statistics by the Statistics Korea, and the State of Urban Planning Report by the Ministry of Land, Infrastructure, Transport, and Tourism.

Although prior studies provided COVID-19 risk factors, none identified COVID-19-vulnerable locales associated with SES and COVID-19-specific epidemiological factors with acceptable generalizability and methodological capacity. Current COVID-19 studies relying on health disparity measures use arbitrary SES variables based on researchers’ preferences, irrespective of their COVID-19 relevance. Consequently, the SES measures across these studies are incomparable, limiting their usefulness. To address this, we integrated Coleman’s Social Theory and Blumenshine’s mechanistic framework (Figure 1), which formulates a universal SES definition and SES indicator selection mechanistically/causally relevant to the COVID-19 health outcome. This approach can inform public health interventions to alleviate SES factor-related COVID-19 risk.

Since SES and epidemiological data cover an expanse of highly intercorrelated variables, the composite SES index, derived from multiple unique SES variables help garner the most explanatory information from the contributing indicators. A single universal composite measure, like the commonly-used area-deprivation index [10], is limited to controlling SES effects as confounders, but not when the study’s goal is assessing the effect of multiple SES determinants on health outcome (e.g., COVID-19 in this study). Therefore, a multiple composite SES index approach helps quantify each composite SES index’s effect on COVID-19 [11].

Thus far, one study used multi-scale Geographically Weighted Regression (GWR) to map the US COVID-19 incidence rate, while accounting for selected SES variables (median household income, income inequality, percentage of nurse practitioners, and black female population) [12]. Since multi-scale GWR doesn’t fit a beta distribution typical for infectious disease rates [13], we recommend Geographically Weighted Negative Binomial Regression (GWNBR) to improve methodological accuracy. GWNBR directly uses discrete count data without further transformation, and is robust in the overdispersion, spatial/temporal clustering and false-positives [14,15].

Globally, the COVID-19 pandemic emerged in waves with country-specific mitigation strategies producing sharp declines. To improve public health interventions by precision targeting of high-risk locales, this study identified key SES and epidemiological risk determinants and their geographic distribution. We chose South Korea because its COVID-19 incidence data presented extremely high overdispersion, temporality, and spatial clustering, being more complex than typical infectious disease data. This allowed us to check our framework’s functionality to address the dramatic spatial and temporal dynamic of COVID-19 [16]. Our study’s goals were to; (1) provide methodological framework for identifying COVID-19-vulnerable locales associated with SES and epidemiological determinants; and (2) operationalize the framework using South Korean data to demonstrate its value.

## 2. Materials and Methods

### 2.1. Study Design and Population

We used COVID-19 incidence data from January 20 through July 1, 2020, released from the Korea Centers for Disease Control and Prevention (KCDC) [17] and prepared by the DS4C project [18]. The dataset is based on the report materials of KCDC and local governments from this time period. These data are available under the KOCL (Korea Open Government License). Analytical data consisted of 11,811 COVID-19 cases aggregated by 250 districts (Table S1) aligned to SAS’s South Korean geographic matrix. Since the data were unavailable for Daegu’s subparts, we estimated the incidence from KCDC’s press release cluster reports.

### 2.2. Conceptual Model

Figure 1 shows the Coleman-Blumenshine Framework (CBF) refined approach, based on Coleman’s Social Theory and Blumenshine’s mechanistic framework [9,19]. The model defines SES as a function of material, social, and human capitals [8,19] and emphasizes pathways by how SES indicators differentially increase SARS-CoV-2 exposure and susceptibility to developing COVID-19 [9]. Based on the CBF model and COVID-19 risk factors literature [1,20,21,22,23], we identified seven area-level health and SES factors that determined the SARS-CoV-2 exposure level and the likelihood of developing COVID-19 after exposure.

### 2.3. SES Measurement and Epidemiological Factors

All SES and epidemiological-related data were retrieved from the Korean Statistical Information Service’s (KOSIS) online data archive [25]. KOSIS offers a convenient one-stop service for South Korea’s major domestic statistics. Table S2 presents the data sources used for SES measurement. Table S3 shows 24 data items out of 124 candidates relevant to the seven health/SE areas. We used an independent variable proxy for *education*, and by Principal Component Analysis (PCA) created six thematic composite indices: *healthcare access*, *health behaviour*, *crowding*, *area morbidity*, *difficulty to social distancing*, and *population mobility*. Factors were computed as linear combinations of the original variables selected for each health/SE theme. We used the first component scores [26] in calculating the composite scores since they explained the largest data variation. Then we computed each variable’s weight by dividing each factor score by the sum of all variable factor scores as,

(1) $$Weight_{i}=Score_{i}/\sum_{i=1}^{p}Score_{i}$$

where *i* relates to each theme’s variable and *p* is the number of each theme’s variables. Each thematic composite index was computed as the weighted average for all 250 district values. For example, the composite index for *Health behaviour* was calculated as:

(2) $$\mathrm{Health}{\mathrm{behaviour}}_{k}\;=\;0.438\times \mathrm{obesity}\;\mathrm{by}\;{\mathrm{measurement}}_{k}\;+\;0.429\times \mathrm{alcohol}\;{\mathrm{drinking}}_{k}\;+\;0.100\times \mathrm{current}\;{\mathrm{smoking}}_{k}\;+\;0.033\times \mathrm{self-reported}\;{\mathrm{obesity}}_{k}$$

where *k* is the original variable’s value for district *k*. Note that weights sum to 1 (0.438 + 0.429 + 0.100 + 0.033 = 1). Six thematic composite indices and an individual proxy for education (percentage of high school educated people) were used in the final models as independent variables.

*The ratio of the 25th percentile and each theme’s maximum value was for healthcare access: 2.5; health behaviour: 1.2; crowding: 1.9; area morbidity: 1.3; education: 1.5; difficulty to social distancing: 3.0; and population mobility: 10.3.2.4. Statistics*

Our model outcome was the confirmed case counts of COVID-19 aggregated by 250 districts. Global negative binomial regression (GNBR) and GWNBR [14] computed relative risk of COVID-19 associated seven area health/SE themes.

### 2.4. Global Models

GNBR models calculated relative COVID-19 risk for the entire study period and each pandemic phase. The global model was set as,

COVID-19 = exp (β_0_ + β_1_*healthcare access* + β_2_*health behaviour* + β_3_*crowding* + β_4_*area morbidity* + β_5_*education* + β_6_*difficulty to social distancing* + β_7_*population mobility* + ε (3)

where, β_0_,…, β_n_ were the intercept and regression coefficients, whereas ε was the model random error.

### 2.5. Local Spatial Models

We used Gaussian GWNBR to model discrete count data and handle overdispersion issues. GWNBR computed parameter estimates for all districts following,

(4) $$y_{j}\sim NB\left[t_{j}\exp\left(\sum_{k}\beta_{k}\left(u_{j},v_{j}\right)x_{jk}\right),\alpha \left(u_{j},v_{j}\right)\right]$$

where ($u_{j},\;v_{j}$) are the locations (coordinates) of the data points *j*, for *j* = 1,…, *n*. The models empirically computed bandwidth, via the cross-validation criterion, and achieved minimal Akaike’s information criterion (AIC) as,

(5) $$CV=\sum_{j=1}^{n}{\left[y_{j}-{\hat{y}}_{\neq j\left(b\right)}\right]}^{2}$$

where ${\hat{y}}_{\neq j\left(b\right)}$ is the estimated value for point *j*, omitting the observation *j,* and *b* is the bandwidth. The likelihood of false-positives was corrected by the method of da Silva and Fotheringham [27].

All statistical analyses including specific macro programs for spatial weight matrices and GWNBR models were implemented using SAS (version 9.4). Missing data (2%) were excluded from the analyses.

## 3. Results

**Figure 2:**
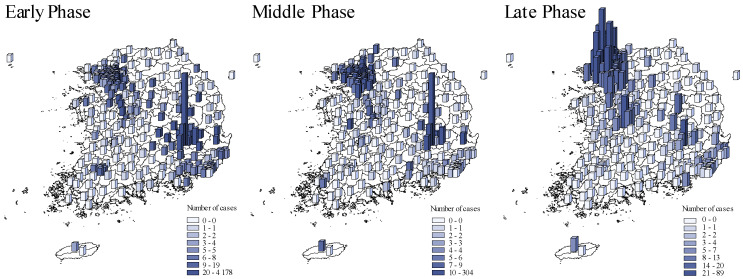
The spatial distribution of COVID-19 cases across pandemic phases. Early phase: from 20 January to 20 March 2020. Middle phase: 21 March to 15 April 2020. Late phase: 16 April to 1 July 2020. The blue shades and bar heights both indicate the number of COVID-19 cases during each in each district.

Figure 2 compared the spatial COVID-19 distribution across pandemic phases. The initial outbreak wave occurred in Daegu which then spread to Gyeongsangbuk-do and surrounding provinces in the early phase [22]. The second wave occurred in Seoul and its surrounding metropolises, Ulsan and Busan, and Gyeonggi-do province in the late phase of the pandemic.

### Global and Local Spatial Models

Throughout the entire study period model, GNBR suggested that the COVID-19 risk associated with increased risky *health behaviour, area morbidity*, and *difficulty to social distancing* (Table 1). Inverse associations indicate an increased COVID-19 risk with reduced *healthcare access*, lower *education*, and increased efflux in *population mobility*. No substantial risk was associated with *crowding*.

**Figure 3:**
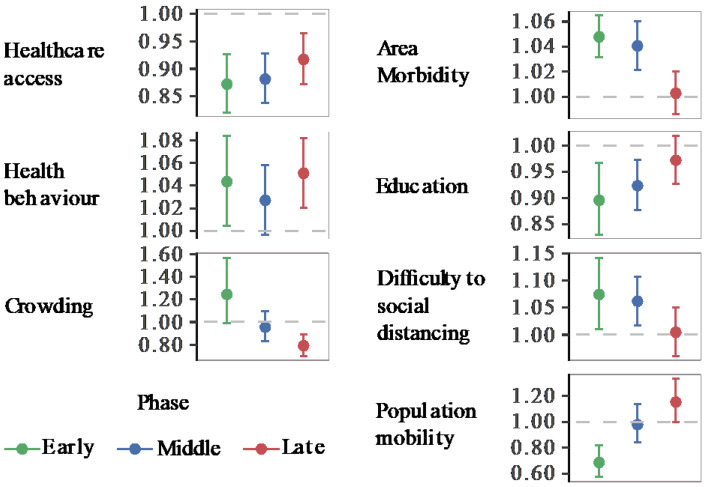
Relative Risk of COVID-19 associated with area health and SES determinants (GNBR models). Each panel shows the relative risk and its 95% confidence intervals associated with each thematic area. Colours represent the pandemic’s early (20 January to 20 March 2020), middle (21 March to 15 April 2020), and late phases (16 April to 1 July 2020). The dashed line shows the reference level (1). Values over or below the reference line indicate statistically significant results at α = 0.05. Corresponding *p*-values can be found in Table S5.

**Figure 4:**
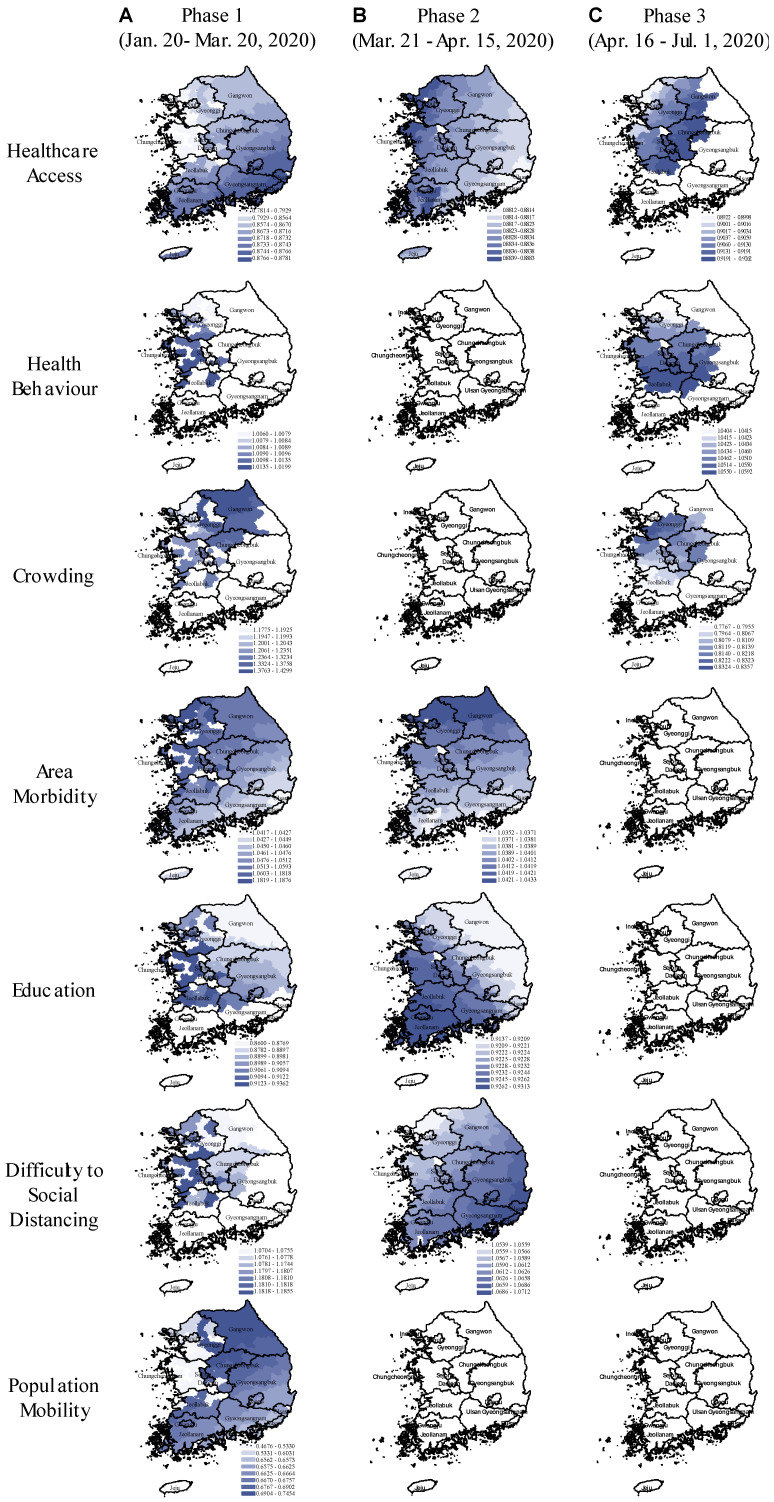
Spatial variation in the relative risk of COVID-19 associated with area-health and SES themes in the early, middle, and late phases of the pandemic (GWNBR models). In the maps, the blue colour gradient corresponds with larger (darker) to lower (lighter) relative risk. Areas in white indicate the relative risks are statistically not significant (α = 0.05). The figure consists of (**A**–**C**) columns respectively referring to the pandemic’s early (20 January to 20 March 2020), middle (21 March to 15 April 2020), and late phases (16 April to 1 July 2020).

We implemented global and local spatial models for the early, middle, and late pandemic phases. Figure 3 presents the relative risk of COVID-19 with its 95% CI from GNBR models, and Figure 4, the relative risk spatial distribution from GWNBR associated with seven thematic areas by pandemic phases. Table S4 provides more details on the stratified GNBR models. GWNBR fit data better than the global model given smaller AIC for the middle and late phases, respectively, (AIC_gwnbr_~1034 vs AIC_gnbr_~1044, AIC_gwnbr_~1038 versus AIC_gnbr_~1074) except for the early phase of the pandemic (AIC_gwnbr_~3533 vs AIC_gnbr_~1527). This reflects the large spatial cluster emerging from Daegu church [22,28] activities during the early phase that subsequently spread to its neighbouring districts. The GNBR and GWNBR model results agreed across all pandemic phases. In the early phase, lower *healthcare access* and *education*, and increased risky *health behaviour*, *area morbidity*, *difficulty to social distancing*, and *population mobility* associated with higher COVID-19 risk. The *crowding-associated* risk was not significant in GNBR. In the middle phase, *healthcare access, area morbidity, education, and difficulty to social distancing* remained important risk determinants. In the late phase, only *healthcare access, health behaviour*, and *increased crowding* significantly determined the COVID-19 risk.

GWNBR created early phase maps showing higher risk in non-contiguous districts (Figure 4A). This higher risk reflected virus transmission in the initially affected districts before spreading over larger areas.

During the early phase, we found protective effects of improved healthcare access, higher education and outbound population mobility, whereas, the disease risk was increased in the districts with higher risky health behaviour, area morbidity rate and difficulty to social distancing.

In the middle phase (Figure 4B), only healthcare access, area morbidity rate, education and difficulty to social distancing remained as the key risk-determinants with the same directions but reduced strengths. Spatial shifts from the early phase were from the northwest toward the capital and southwest regions.

In the late phase (Figure 4C), healthcare access, risky healthy behaviour and area crowding were primary risk-determinants. We observed the protective effect of improved healthcare access, while risky health behaviour was the significant risk factor. In contrast to the early phase, we found higher disease risk in more crowded districts. In the late phase, risk-determinants concentrated around the capital and middle regions. Taking the type of risk-determinants and their spatial distributions across the three phases together, our results showed that pandemic had evolved from lower to higher density areas. This led to the second wave that emerged in Seoul and its surrounding areas.

We observed noticeable spatial shifts in the risk determinants over the study period (Figure 4). *Difficulty to social distancing* increased COVID-19 risk in the capital and middle regions in the early phase which then shifted to the country’s southeast part in the middle phase. *Area morbidity*-associated risk was concentrated in the western part which then gradually shifted north in the middle phase. *Education*-associated risk was higher in the west in the early phase until it shifted southwest in the middle phase. *Population mobility* elevated COVID-19 risk only in the early phase for South Korea’s northern, eastern, and western parts.

We investigated the correlations between all pairs of composite indices (Table S6). The largest Pearson’s r was 0.603 between *healthcare access* and *area morbidity*. We verified no multicollinearity given that the model’s standard error of both *healthcare access* (0.025) and *crowding* (0.09) were small. For a direct comparison between non-spatial and spatial models, GNBR and GWNBR were carried out with the same variables and stratified by the same periods (NBR: Figure 3, and GWNBR: Figure 4). AIC and dispersion coefficients were used to compare the models’ goodness of fit.

## 4. Discussion

This study provided a methodology to map COVID-19 risk associated with multiple SES and pandemic-specific epidemiological factors with high geographical granularity. We refined a social theories-enhanced CBF model to guide our variable choices. This model warrants the potential variables identified from the COVID-19 risk factor literatures encompass the conceptual domains of (material, human, and capital) SES and plausible to cause differential exposure, susceptibility, and disease severity. The simultaneous influence of multiple SES determinants defines disease risk. By creating multiple composite SES indexes using PCA, this study clarified whether certain SES determinants independently contributed to the COVID-19 risk over and above other SES factors.

GWNBR created a continuous surface of relative COVID-19 risk for all 250 districts associated with area-health and socioeconomic determinants by the pandemic phases (Figure 4). Our findings are consistent with individual and population-level studies that reported elevated COVID-19 risk associated with less *healthcare access* [29], and *education* [30,31], and more *risky health behaviour*, *crowding*, *specific comorbidities* [1,20,23], *difficulty to social distancing* [2,21] and *population mobility* [32]. Our study’s high internal validity was shown since the GNBR and GWNBR results agreed except for *crowding* in the early phase.

Our approach captured statistically and noticeably high spatial variation by pandemic phases for all themes, consistent with the reported pattern of COVID-19 distribution in the country [22,28]. Since its first confirmed case on January 20th, 2020, South Korea experienced two major outbreak waves in Daegu and Seoul, and the surrounding Gyeonggi-do province, respectively, in February (early phase) and May 2020 (late phase).

After the first confirmed case, the KCDC invoked four-level alert for the public’s emergency awareness (blue-attention, yellow-caution, orange-alert, red-serious) commensurate with the number of new confirmed cases [http://www.koreabiomed.com]. The epidemic’s initial wave in Daegu, caused by the local church activities, triggered the country-wide directives of hospital-based isolation/quarantine, contact tracing followed with free testing and treatment, strengthening medical centres for rapid diagnostics, emergency medical responses, and treatment aids [22]. These specific measures along with high public adherence to the school and business closures, personal hygiene, and social distancing significantly dropped the case counts by mid-March. The second wave erupted in May when non-essential businesses reopened [28] in Seoul, which spread to its surrounding metropolises, Ulsan and Busan, and Gyeonggi-do.

*The types of risk determinants changed over the pandemic phases*. Analysis stratified by periodic phases found that the initially high risk in the early period gradually decreased except healthcare access, health behaviour and crowding- associated risk, which increased in strength and concentrated in the capital and its surrounding provinces in the late phases (Figure 3 and Figure 4). Risk reductions could be explained by the impact of effective control measures that lowered the risk associated with these determinants and drop in an effective reproduction number (Re), as the number of infection-susceptible people decreased over time [33].

In the early phase, all health/SE themes were statistically significantly associated with COVID-19 incidence. As anticipated, there was no excess risk at Daegu and its surrounding areas since the abnormally high spike of COVID-19 cases was caused by local church’s activities [22,28] without relevance to the local area’s social status.

The increase in health behaviour-associated risk is consistent with reports showing greater risk with poor emotional health [30], smoking [34], and obesity [1,35]. Individual patient-level COVID-19 risk-factors analysis Lusignan et al.,2020 [1] reported smoking was a protective factor. However, the authors warned that the low proportion of current smokers in their study sample (11.4%), resulted in a wide confidence interval of the reported odds ratio, 0.59 (0.42–0.83). This increases the uncertainty of their result. Greater COVID-19 risk among the people with lower education has been explained as a reduced awareness of disease risk and low-income to obtain education. This relationship was clearly seen in our results as higher education associated with lower COVID-19 incidence. The difficulty to social distancing in this study directly reflected the inability to afford unemployment possibly resulting in the reduced exertion of protective measures [30,31]. The risks associated with social distancing and area-morbidity peaked in the study’s early phase appeared to reduce in the middle phase, and completely remediated in the study’s late phase.

In the middle phase, all of the previous risk factors except for risky health behaviours, population mobility, and crowding were high. The middle phase’s lessened risk associated with risky health behaviours, population mobility and crowding may reflect the impact of the Prime Minister’s declaration. This implemented active interventions for social distancing, community health education, testing with local contact, tracing, and hospital-based or self-isolation during March’s first weeks.

Notably, the late phase findings are consistent with the risk factors reported associated with the second wave in early May. During our study’s late phase, South Korea scaled up free testing and treatment through its existing health care centres [28,36], which may have improved healthcare access a key measure for combating COVID-19. Our finding that healthcare access exerts a stronger protective effect in the late phase compared with the earlier phases supports this. Our findings of increased risk associated with risky health behaviours may have captured behavioural fatigue at a population-scale in response to the country’s multiple quarantine period extensions [37] that likely were exacerbated by entertainment business re-openings (i.e., night clubs, karaoke) in early May. Elevated risks associated with increased crowding in the study’s late phase reflects the outbreak’s second wave, which occurred in South Korea’s most crowded region: Seoul and its surroundings (Figure 3).

*Spatial variation in the SES-related risk factors across the pandemic phases potentially reflect the geography-specific control measures and/or the differential public response to the measures.* GWNBR models revealed the pandemic phase-specific spatial variation for all health/SE themes except for population mobility which was not significant beyond the early phase. This may indicate that the effectiveness of the control measures varied over time potentially due to differential interventions or public response across the municipal districts. Our findings may also indicate a dynamic change in population vulnerability throughout the pandemic “a person not considered vulnerable at the outset of a pandemic can become vulnerable depending on the policy response” as a Lancet editorial stated [38].

The factors increasing our recommended framework’s robustness include: (1) SES measurement and relationship conceptualization of the exposure (health/SE themes) and outcome (COVID-19 incidence) based on the refined conceptual framework; (2) joint use of conceptual and statistical modelling; (3) complementary use of global and local spatial statistics; and (4) stratified analysis by pandemic phases that enable us to capture the spatial variation over pandemic phases. However, this methodological framework relies on carefully collected country-specific data.

Our study is subject to ecological fallacy inherent to the study design. However, our empty hierarchical mixed model accounting for the individual and district-level data shows that 61% of the COVID-19 incidence distribution variation was explained by the district-level factors, leaving 39% of the variability for an explanation by individual factors.

We verified that the data estimation for Daegu city subparts did not affect the study results. The comparison of the intercept, standard error, relative risk and *p*-value between the models with and without the estimated data showed that the intercept and standard error were diminished by 2.2% and by 9.2%, respectively in the models, including estimated data [each calculated by 100 × (−12.29 − (−12.56))/−12.29 and 100 × (1.88 − 2.95)/1.88, respectively]. A significance level change was observed for none of the model estimates, except for *crowding.* The *p*-value changed from ~0.06 to ~0.04 when the estimated data were excluded. However, the *crowding-associated* risk remains significant at *p* = 0.1. Model details are provided in Table S4. To assess the periodic trend in the relative COVID-19 risk associated with SES factors, we conducted stratified analyses by the early, middle, and late phases corresponding with 20 January–20 March, 21 March–15 April, and 16 April–1 July 2020.

Population-based COVID-19 studies are prone to response bias, which would not exist if everybody was tested. However, multiple factors determine testing coverage, therefore, the number of confirmed cases, such as easy access for testing and its accuracy [39], contact tracing strategies [40], under-testing of asymptomatic patients [41]. Also, psychological factors, a fear of COVID-19 [42], risk perception [43,44,45], and stigma related testing avoidance [46] impact testing rate. Potential bias in this study is expected to be low given South Korea’s anti-pandemic strategies. Importantly, the country’s COVID-19 relief programs supported with 15 billion Korea won, dedicated to support vulnerable populations may have reduced the potential testing disparity by socioeconomic status. All Koreans and foreigners were entitled to free testing and treatment, while testing access was more convenient through an extended number of rapid diagnostic centres and testing prompts through mobile phones. Contact tracing-based testing increases the likelihood of capturing asymptomatic cases. Korean tracing system has been reported as the global best practice lending to its advanced information technology system and data extensions through large consumer and healthcare databases (global positioning system, credit card transactions, closed-circuit television and medical facility use records) [47,48]. Testing avoidance from fear of stigma [46,49] would likely have affected the early period of the analysis, which strongly reflected the abnormally high spike of cases in Daegu city traced to the local church. The city has not disclosed the data with necessary granularity for a further investigation of this matter, as of writing. However, given these factors would likely result in under-estimation of confirmed cases, any bias in our results should be toward the null.

## 5. Conclusions

The demonstrated methodology guides to design of multiple-determinant targeted interventions and pinpoint high-risk locales to remediate the excess COVID-19 risk attributable to socioeconomic disadvantages. Overall, our work has demonstrated that the anti-pandemic measures taken by the South Korean government were effective.

1. The completely remediated risk associated with area-morbidity and difficulty to social distancing is likely to be explained by the country’s emergency relief programs that targeted vulnerable individuals with socioeconomic disadvantages: Foreign workers, homeless, poor urban residents, disabled people, and elderly. The assistance programs provided free testing, financial support, food assistance, health check-up visits, as they acknowledged excess hardship in adhering to social distancing rules because of inability to afford unemployment.
2. The observed overall protective effect of improved healthcare access and higher education in our study support the rationale behind the country’s primary anti-pandemic agenda to strengthen healthcare facilities for rapid diagnostic and therapeutic services, combined with actionable health promotion rules which reportedly gained high public compliance.
3. However, we found risky health behaviour was a persistent risk factor during both major outbreaks in Daegu and Seoul. Elevated crowding associated risk coincided with the Seoul outbreak, as anticipated.
4. Persistently high-risks associated with health behaviour and crowding, combined with the reduced protective effect of healthcare access and education in the study’s late phase may corroborate the finding that a prolonged pandemic induces adherence fatigue and lessened risk perception [31,37].

South Korean public health interventions have been discussed in detail elsewhere [28] and we endorse the country’s anti-pandemic interventions as guidance to international policymakers. The main highlights were: (1) Targeting vulnerable locales to COVID-19 and aiming to address multiple risk factors considering emergency relief programs to provide financial support, food assistance, health check-up visits; (2) implementing social distancing while assisting individuals with difficulty to social distancing. Social distancing measures may include school/business closures, hospital-based or self-isolated quarantine; (3) improving healthcare access for expanded testing and treatment with priority health services made available to the individual with extenuating medical conditions; (4) strengthening existing healthcare facilities and extending rapid diagnostic centres to enable easily accessible and free testing; (5) enhancing case identification capacity through information technology, such as contact tracing, mobile phone-based testing prompts and general risk alerts, rapid case-isolation by automated test result delivery to the testee’s mobile phones.

We emphasize the importance to anticipate adherence, behavioural, and mental fatigue over the course of a prolonged epidemic. In the latter phase of the epidemic, we recommend paying intensified attention to the urban and highly crowded areas to prevent a potential outbreak as well as promoting creative social networking solutions (drive-through services, virtual social events, telehealth, etc.) and ensuring emerging vaccine accessibility for the socially disadvantaged population [50].

We intend that this framework can be replicable to both, international researchers and policymakers, in order to enable rapid pandemic responses. As socioeconomic disparity is a global problem, nationwide programs with an intensified focus on the vulnerable populations at excess risk to pandemic ensure the efficacy and efficiency of pandemic alleviation efforts.

Future research should assess the mortality, and mortality and incidence ratio as a crude surrogate for survival using the same study design and methodology. Understanding the impact of socio-economic and epidemiological risk factors on mortality compared with incidence would clarify the extent of the potentially preventable deaths through modifications of the assessed risk factors. The overall and spatial disparity between mortality, incidence, and mortality/incidence ratio would inform where intensified public health and intensified healthcare services are needed.

## Figures and Tables

**Table 1:** Parameter estimates and 95% CI of the relative risk of COVID-19 associated with health and SES determinants during the entire study period (20 January–1 July 2020).

Health/SE themes	Estimates	Relative Risk (95% CI)	*p*-Value
Healthcare access	−0.13	0.88 (0.84–0.92)	<0.0001
Health behaviour	0.04	1.04 (1.01–1.07)	0.019
Crowding	0.05	1.05 (0.89–1.25)	0.545
Area morbidity	0.04	1.04 (1.03–1.06)	<0.0001
Education	−0.09	0.91 (0.86–0.97)	0.002
Difficulty to social distancing	0.06	1.06 (1.01–1.12)	0.017
Population mobility	−0.22	0.80 (0.69–0.93)	0.003
Dispersion ^a^	2.49		
AIC	1850		

^a^ The variance of a negative binomial distribution; Abbreviations: Akaike’s information criterion (AIC), a measure of goodness of model fit; confidence interval (CI); socioeconomic (SE).

## Data Availability

The COVID-19 incidence data used in this study is available through a publicly accessible repository https://www.kaggle.com/kimjihoo/coronavirusdataset. Socioeconomic and epidemiological datasets used in this study can be found at https://KOSIS.kr.

## Supplementary Materials

The following are available online at https://www.mdpi.com/1660-4601/18/2/604/s1, Table S1 Confirmed cases of COVID-19 diagnosed during 20 January–1 July 2020 in South Korea by pandemic phases, Table S2: Data sources reviewed and used for SES measurement, Table S3: PCA details showing factor scores with their weights, and selected area health and SES variables and thematic composite indices, Table S4: GNBR model estimates with and without the estimated data for Daegu’s subparts throughout study period (20 January through 1 July 2020), Table S5: Parameter estimates and the Relative Risk of the COVID-19 incidence associated with health and SES determinants by three time periods corresponding with the early, middle and late phases, Table S6: Matrix table of Pearson’s correlation coefficients (r).
