# Supplementary material for: Precision Mapping of COVID-19 Vulnerable Locales by Epidemiological and Socioeconomic Risk Factors, Developed Using South Korean Data
Source: Int J Environ Res Public Health. 2021 Jan 12;18(2):604. doi: 10.3390/ijerph18020604 (PMC7828122; doi:10.3390/ijerph18020604)
Supplement: Supplementary file 1 [file ijerph-18-00604-s001.pdf]

Supplementary Materials

Table S1. Confirmed COVID-19 cases diagnosed during January 20 - July 1, 2020 in South Korea

Province	Early phase	Middle phase	Late Phase
Busan	99	18	24
Chungcheongbuk	34	11	12
Chungcheongnam	85	17	28
Daegu	6358	529	35
Daeguon	21	17	80
Gangwon	30	20	13
Gwangju	48	2	3
Gyeonggi	324	325	567
Gyeongsangbuk	1098	111	45
Gyeongsangnam	86	26	17
Incheon	38	47	253
Jeju	3	9	6
Jeollabuk	8	9	4
Jeollanam	4	10	0
Sejong	34	10	7
Seoul	298	278	656
Ulsan	32	8	15

COVID-19 cases are sorted by South Korea's provinces (comprised of 250 districts) and the pandemic phase. Early phase: January 20 to March 20, 2020; Middle phase: March 21 to April 15, 2020; Late phase: April 16 to July 1, 2020.

Table S2. Data sources reviewed and used for SES measurement

Data sources	Data items
Korean Community Health Survey 2018, Korea Centres for Disease Control and Prevention	(%) people with obesity, by measurement<br>(%) people drunk alcohol <1 per month<br>(%) people who currently smoke<br>(%) people obese, self-reported<br>(%) of people who used health care last year<br>(%) people who could not use healthcare when needed last year<br>(%) people with depression based of PHQ-9[a] screening<br>(%) of the person who is all aligned for the early symptoms of stroke<br>(%) of the person on insulin and other treatment specific to diabetes mellitus
Health Insurance Statistics 2018, National Health Insurance Corporation	(%) people with health insurance
Disability Status 2018, Ministry of Health and Welfare	(%) people with severe disability
Death Cause Statistics 2018, National Statistics Agency	Age adjusted mortality rate due to neoplasm<br>Age adjusted mortality rate due to circulatory system disease<br>Age-adjusted mortality rate from infectious parasitic diseases<br>Overall, age-adjusted mortality rate<br>Age adjusted mortality rate due to respiratory diseases
Korean Census Bureau 2015	(%) people with high school education<br>(%) of foreign registered people<br>Number of people per household
Office of Statistics 2015, Regional Statistics	GDP per capita in million won
Internal Migration Statistics 2018, Statistics Korea	Internal net migration between regions
State of Urban Planning 2018, Ministry of Land, Infrastructure, Transport and Tourism	Area per capita<br>Urban area per capita

[a]PHQ-9: Patient Health Questionnaire -9, standard survey tool.

Table S3. PCA details showing factor scores with their weights, and selected area health and SES variables and thematic composite indices

Health/SE themes	Selected variables from national surveys	PCA Factor	Weights	Quartiles of composite indices			
				25th pctl	Median	75th pctl	Max
Healthcare access	Healthcare utility rate	0.892	0.448	18.0	21.0	29.6	44.2
	Insurance coverage rate	0.869	0.437				
	Healthcare needs met when needed	0.226	0.115				
Health behaviour	% people with obesity, by measurement	0.955	0.438	41.6	44.0	45.7	49.9
	% people drunk alcohol, < 1/month	0.936	0.429				
	% people who currently smoke	0.220	0.100				
	% people obese, self-reported	0.069	0.033				
Crowding	[a] Area per capita	0.900	0.402	11.6	12.9	14.6	22.6
	[a] Urban area per district	0.734	0.329				
	N of households per capita	0.602	0.269				
Area morbidity	Overall, AAMR	0.919	0.190	88.1	96.7	102.7	116.9
	Respiratory, AAMR	0.838	0.173				
	Circulatory, AAMR	0.743	0.153				
	Infectious and parasitic diseases, AAMR	0.702	0.145				
	% people with severe disability	0.689	0.142				
	% people on diabetes treatment	0.483	0.099				
	% people with stroke symptoms	0.436	0.090				
	% people with mental health diseases	0.020	0.008				
Difficulty to social distancing	% people living in apartment buildings	0.794	0.286	9.2	13.3	15.4	27.6
	% workers in retail services	0.749	0.269				
	N of students per class, high school	0.677	0.244				
	% workers in health and social services	0.558	0.201				
Population mobility	Net migration between districts	0.732	0.500	0.7	1.1	1.9	7.2
	% foreign residents in the area	0.732	0.500				

The factor scores and weights of each contributing variable associated with the first PCA-identified component and the quartiles of the health/SE themes are shown. Education is not included since it is already a single variable. Abbreviations: Principal component analysis (PCA); Socioeconomic (SE); age-adjusted mortality rate (AAMR). Superscripts: [a] km^2.

Table S4. GNBR model estimates with and without the estimated data for Daegu's subparts throughout study period (January 20 through July 1, 2020)

	Included estimated data				Excluded estimated data			
Health/SE themes	Estimate[a]	SE	RR (LCL - UCL)	P-value	Estimate	SE	RR (LCL - UCL)	P-value
Area-morbidity	0.05	0.01	1.05 (1.03 - 1.06)	<.0001	0.04	0.01	1.04 (1.03 - 1.06)	<.0001
Education	-0.11	0.04	0.90 (0.83 - 0.97)	0.005	-0.12	0.04	0.89 (0.82 - 0.96)	0.003
Crowding	0.22	0.12	1.25 (0.99 - 1.57)	0.06	0.27	0.13	1.30 (1.01 - 1.69)	0.04
Difficulty to social distancing	0.07	0.03	1.07 (1.01 - 1.14)	0.02	0.08	0.03	1.08 (1.02 - 1.15)	0.01
Population mobility	-0.38	0.09	0.69 (0.57 - 0.82)	<.0001	-0.34	0.09	0.71 (0.59 - 0.85)	0.0002
Healthcare access	-0.14	0.03	0.87 (0.82 - 0.93)	<.0001	-0.13	0.03	0.88 (0.82 - 0.93)	<.0001
Health behaviour	0.04	0.02	1.04 (1.00 - 1.08)	0.03	0.04	0.02	1.04 (1.00 – 1.08)	0.05
Dispersion	3.68				3.69			
AIC	1527				1425			

Superscripts: [a] Parameter estimates; Abbreviations: Standard error (SE); Relative Risk (RR); lower boundary of 95% confidence interval (LCL); upper boundary of 95% confidence internal (UCL); Global negative binomial regression (GNBR); Akaike's information criterion (AIC).

Table S5. Parameter estimates and the Relative Risk of the COVID-19 incidence associated with health and SES determinants by three time periods corresponding with the early, middle and late phases

	Early phase			Middle phase			Late phase		
	**Estimate**	**RR (LCL - UCL)**	**P-value**	**Estimate**	**RR (LCL - UCL)**	**P-value**	**Estimate**	**RR (LCL - UCL)**	**P-value**
Healthcare access	-0.14	0.87 (0.82 - 0.93)	<.0001	-0.13	0.88 (0.84 - 0.93)	<.0001	-0.09	0.92 (0.87 - 0.96)	0.001
Health behaviour	0.04	1.04 (1.00 - 1.08)	0.028	0.03	1.03 (1.00 - 1.06)	0.080	0.05	1.05 (1.02 - 1.08)	0.001
Crowding	0.22	1.25 (0.99 - 1.57)	0.058	-0.05	0.96 (0.83 - 1.10)	0.512	-0.23	0.79 (0.70 - 0.89)	0.000
Area morbidity	0.05	1.05 (1.03 - 1.06)	<.0001	0.04	1.04 (1.02 - 1.06)	<.0001	0.003	1.00 (0.99 - 1.02)	0.729
Education	-0.11	0.90 (0.83 - 0.97)	0.005	-0.08	0.92 (0.88 - 0.97)	0.003	-0.03	0.97 (0.93 - 1.02)	0.223
Difficulty to social distancing	0.07	1.07 (1.01 - 1.14)	0.021	0.06	1.06 (1.02 - 1.11)	0.006	0.005	1.00 (0.96 - 1.05)	0.843
Population mobility	-0.38	0.69 (0.57 - 0.82)	<.0001	-0.02	0.98 (0.84 - 1.14)	0.791	0.14	1.15 (0.99 - 1.33)	0.061

Early phase: January 20 to March 20, 2020; Middle phase: March 21 to April 15, 2020; Late phase: April 16 to July 1, 2020. Abbreviations: Relative Risk (RR); lower boundary of 95% confidence interval (LCL); upper boundary of 95% confidence internal (UCL).

Table S6. Matrix table of Pearson's correlation coefficients (r)

	**Healthcare access**	**Health behaviour**	**Crowding**	**Area morbidity**	**Education**	**Difficulty to social distancing**	**Population mobility**
Healthcare access	1						
Health behaviour	0.547	1					
Crowding	0.568	0.124	1				
Area morbidity	0.604	0.534	0.415	1			
Education	0.127	0.390	-0.080	0.510	1		
Difficulty to social distancing	-0.032	0.370	-0.047	0.311	0.315	1	
Population mobility	-0.096	0.086	-0.019	0.018	0.163	0.129	1
